# The Book World of Medicine and Science

**Published:** 1899-02-25

**Authors:** 


### The Book World of Medicine and Science.

A Handbook of Midwifery, Specially Designed for the Use of Candidates for the Certificate of the Obstetrical Society of London. By Alfred S. Gubb, M.D., &c. (Aberdeen: James G. Bisset. 1898. Price 4s., nett.

This handbook has been specially devised to assist student-midwives and monthly nurses in obtaining a practical if elementary knowledge of the conduct of an ordinary uncomplicated labour, but it contains in addition a general description of most of the complications which may be met with so as to enable the midwife to recognise them and to understand in some sort their bearings upon the progress of the case. The treatment, however, of such complications as necessitate the sending for qualified assistance is left either undescribed or only slightly indicated. The book is likely then to serve very well as a guide to the examination of the Obstetrical Society. The descriptions given of the anatomy of the parts concerned are short and to the point, and by aid of the illustrations are easy to follow, as also are those of the signs of pregnancy and the successive events of labour. The management of an ordinary case having been described, the mechanism of labour and the causes of obstructions are entered upon, the whole problem being made very clear by aid of letterpress and diagrams. After this comes a very important heading, namely, "When to Send for the Doctor?" The answer to this question is practically covered by the following sentence: "Speaking generally, anything unusual occurring during pregnancy or labour should lead the nurse to seek medical assistance;" but the matter is made still more definite by a series of rules which ought to leave no doubt or hesitation in the mind of the midwife as to the circumstances under which she ought to send for help. Taken altogether the book will be found a plain and comprehensible guide to the subject of which it treats. There are, however, a few points in regard to which we think it is open to criticism. We cannot quite bring ourselves to believe in the efficacy of a solution of carbolic acid of the strength of "a teaspoonful to a pint of hot water," *i.e.*, one part in 160 parts, for disinfecting the hands, or of one of 60 minims to the quart, or one in 320 for other purposes. Nor do we think it a nice plan in making a vaginal examination, after anointing the finger with "lard, oil, or vaseline," to "run the tip along the groove between the buttocks until the vulva is reached," &c., and we should have liked to see some sort of directions given as to the care of the infant's eyes in case of signs of inflammation occurring. As to the administration of ergot by a midwife who is only supposed to attend ordinary cases, we should have thought that except in anticipated post partum hæmorrhage it was hardly in place at all. This handbook for the L.O.S. examination, however, seems to take for granted that its administration is within the midwife's province, although with great wisdom it says "ergot is a powerful drug, and should be given with prudence, especially in a primipara, in whom the soft parts are naturally slower to yield, and the exact condition of whose pelvic canal is to a great extent unknown." A list is given of the occasions "when not to give ergot," among which we find "when there is any deformity of the pelvis" and "in transverse and other malpresentations of the fœtus," and at the end of the list it is added, "ergot should never be given during the first stage of labour or when the head is high up near the brim." All of this is most admirable advice, but surely the next sentence ought to cover the whole question so far as midwives are concerned. It runs as follows: "The safest rule is not to give ergot at all until after the child is born." We do not like to think of midwives dabbling in ergot and of their being only restrained from its use by rules "when not to give" it. These, however, are little slips; it is easy in writing a book to forget for whom it is being written.

The London Water Supply: A Retrospect and a Survey. By Richard Sisley, M.D., and Doctor of State Medicine in the University of London. (London: The Scientific Press. 1899. Price 21s.)

This handsome volume, containing, as it does, a full statement of the present condition of the water supply of London, ought to be found useful by that large number of persons who are interesting themselves in the question of how that supply is to be improved. All useful argument should be founded upon fact, and of facts concerning the water supply this book is full. It describes the springs, the rivers, and the wells from which the water is obtained, the pumps by which it is raised, the filters by which it is purified, the reservoirs in which it is stored, and even the pipes by which it is distributed; and all this not in general terms, but in full detail as to each of the various companies engaged in the work. From a literary point of view the greatest interest centres in that part of the book which is devoted to "A Retrospect," in which we find a pleasantly-written account of the manner in which one source of supply after another was brought under requisition to meet the constantly-growing demands for water entailed by the increasing population of London. It is interesting to observe how early in the history of the city complaints were made of the contamination to which the streams flowing into London were subjected, and to note that in the time of Edward I. the question of river pollution was brought before the Parliament which was then sitting at Carlisle. The Earl of Lincoln then complained that the pollution of the Old Bourne (which seems to have run down near the site of Holborn Hill) had become unendurable in consequence of the filth from the tanneries situated on the banks of the stream. Nor is it without interest to note that from the year 1582 until well on in the present century part of the water supply of London was derived from the Thames at London Bridge, where first one, then two, and finally six arches of the bridge were leased to one Peter Morris and his successors, that water wheels might be placed in them, by which to pump up the Thames water. Even in 1821, notwithstanding the great increase of the population above the bridge, it was stated that the "state of pauperism" in which the company found itself was due, not so much to the quality of the water as to the growing height of the houses and the fact that the New River Company could deliver water at a much higher pressure than the old pipes of the London Bridge Company could bear. But then people in those days put up with a good deal, and although it was admitted that the water was frequently foul, they were content to let it stand in the cistern for 24 hours, when it became "finer than any other water that could be imagined." Sentiment clearly had no place in the sanitation of the period.

After the "retrospect" the rest of the book is devoted to a detailed description of the works of the various companies. This, the really useful part of the book, it is impossible to praise too highly. The descriptions are lucid, the maps and plans are admirably printed and set out, and every detail is given which can be required by anyone who wishes to make himself conversant with the very elaborate arrangements which now exist for the production, purification, and distribution of the water supply. It is a far larger business, and has required for its development the exercise of a far greater amount of knowledge and experience and care than some people seem to think, and we cannot imagine a better preparation for useful participation in the discussions which are now imminent in regard to the question of London water supply than a careful study of the facts which are so well presented in this volume.

